# Risk factors associated with aortic calcification in hemodialysis patients

**DOI:** 10.22088/cjim.9.4.347

**Published:** 2018

**Authors:** Alireza Peyro-Shabani, Mehrdad Nabahati, Mohammad-Ali Saber-Sadeghdoust, Mohammad Jafar Soleymani, Farshid Oliaei

**Affiliations:** 1 Student Research Committee, Babol University of Medical Sciences, Babol, Iran.; 2 Cancer Research Center, Health Research Institute, Babol University of Medical Sciences, Babol, Iran; 3 Department of Radiology and Radiotherapy, Babol University of Medical Sciences, Babol, Iran.; 4 Babol University of Medical Sciences, Babol, Iran; 5 Cellular and Molecular Biology Research Center, Health Research Institute, Babol University of Medical Sciences, Babol, Iran

**Keywords:** Dialysis, iPTH, Ca, P, ALK-P, vascular calcification, cardiovascular disease

## Abstract

**Background:**

There are some uncertainties among the risk factors of vascular calcification in the hemodialysis patients. This study was planned to examine the association between abdominal aortic calcification and concerned biochemical parameters in hemodialysis patients.

**Methods:**

In this cross- sectional study, 84 stable hemodialysis patients admitted on hemodialysis section of Shahid Beheshti Hospital in 2013 were enrolled after obtaining informed consent. Pre-dialysis venous blood samples were taken from patients to determine the amount of intact parathyroid hormone (iPTH), alkaline phosphatase (Alk.P), C - reactive protein (CRP), calcium (Ca) and phosphorus (P). Patients underwent abdominal CT scanning and ACI (ACI) was calculated. Statistical analysis was performed using SPSS Version 20. Chi-square, Kruskal Wallis and One Way ANOVA tests were used. P-values < 0.05 were considered significant.

**Results:**

The average age of participants was 50.15±17.03 years (18-83 y/o).A statistically significant correlation was observed between ACI and ALK-P serum levels (p=0.01). It was found that ACI had a significant relationship with phosphorus in women (p=0.01). ALK-P serum levels in men also had a significant relationship with ACI (p=0.02). In addition, there was a significant correlation between ACI and history of cerebro-cardiovascular disease and also duration of dialysis (p=0.004 and 0.0001, respectively).

**Conclusions:**

In patients with longer duration of dialysis, and patients with a history of cardiovascular and cerebrovascular events, ACI levels were significantly higher. ALK-P and phosphorus were correlated with aortic calcification in males and females respectively. No significant correlation was found between iPTH serum levels and aortic calcification.

Cardiovascular disease (CVD) and stroke are the main causes of morbidity and mortality in end-stage renal disease (ESRD). The risk of CVD in ESRD is about 10-20 times more than the normal population (1). The range of vascular changes in patients with ESRD is studied from three aspects: the atheromatous plaques by intima media thickness (IMT), sclerosis and vascular stiffness (compliance reduction) with pulse wave velocity (PWV) and vascular calcification by lateral abdominal radiography or axial CT (2). There are two types of arterial calcification: atherosclerotic lesions with calcium deposits in intimal layer which is associated with fat accumulation, macrophage infiltration and fibrous tissue (3) and medial calcification or Monckeberg that is not common in ordinary people and is usually seen in diabetic or uremic patients. Medial calcification reduces vascular compliance and affects the blood pressure and induces stress on the myocardium and major vessels (4).

On the other hand, secondary hyperparathyroidism occurs at the early stages of kidney failure and can affect other organs such as soft tissues and the bones (5). The relationship between the parathyroid hormone (PTH) secretion disorder and cardiovascular calcification is completely unclear and no clinical studies has systematically checked the parathyroidectomy effect on vascular calcification (5), and it seems that mortality is increased at very high PTH levels (6). In general, it seems that both the upper and lower limits of PTH level can induce vascular calcification. Due to the uncertainty about many risk factors for abdominal aortic calcification, this study was done in the hemodialysis patients.

## Methods

In this cross-sectional study, 84 out of all hemodialysis patients admitted to the Shahid Beheshti hospital in 2013 who had the inclusion criteria were included after obtaining written consent. This study was approved by the Ethics Committee of the Babol University of Medical Sciences. Exclusion criteria were acute infection, known malignancy, active vascular collagen diseases such as rheumatoid arthritis and intake of phosphate binders like sevelamer or aluminum hydroxide during the tests (in cases where the patient used these drugs, it should had been stopped at least a month before the entry into the plan), duration of dialysis less than 3 months, diabetes, being under oral anticoagulant therapy and having a history of parathyroidectomy. Calcium carbonate was continued and because of short term discontinuation of phosphate binders, there was no ethical issue. History of cardiovascular or cerebrovascular events was collected based on history and medical records. Patients were dialyzed with low flux polysulphone dialysers and dialysate calcium of 1.5 mmol / L 3 times a week, each time for 4 hours. Online Kt/V of all patients were over 1.1 .Blood samples of 5cc were taken from patients in the morning before dialysis in the tube containing EDTA anticoagulant to determine the amount of iPTH, CRP, P, Ca and ALK-P. All subjects were fasting and all experiments were performed at the hospital. 

**Figure 1:**
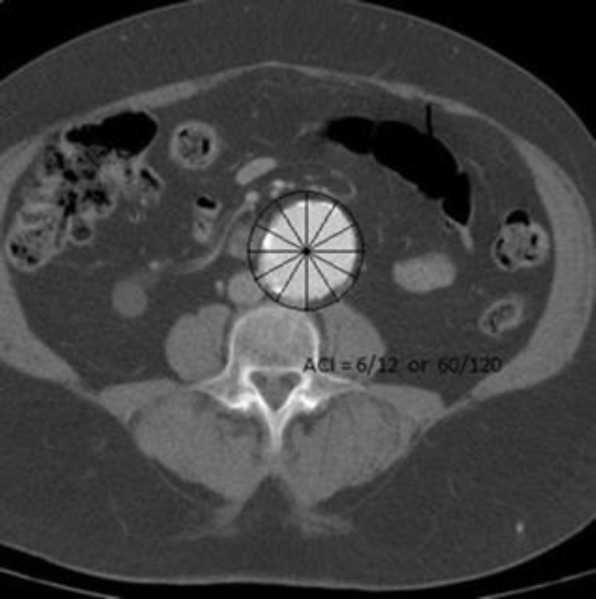
ACI measurement (score 6/12 in this cut)

Blood samples were poured in a dry tube and centrifuged for 10 minutes in 1000cycle/min, the serum was separated and kept at -70°C. All samples were analyzed simultaneously. All patients underwent axial CT scan without contrast on the abdominal aorta. The CT scanner was a multi-slice – 16 (Siemens). Aortic calcification index (ACI) measurement was as following: CT scan without contrast was taken in 10 sections from abdominal aorta between the place of separation of kidney arteries and the bifurcation of abdominal aorta. Each cross section of the aorta was divided into 12 sectors (fig. 1). The sectors in which calcification (with HU more than 100) were seen were counted and then the sum was calculated to obtain ACI score for each patient. ACI of patients was analyzed by a professional radiologist blinded about the patients. 

CT scans and measurement of biochemical indices per person was almost simultaneously done. ACI of patients was divided into three groups: 0-40, 41-80 and 81-120 (sectors) . To collect information, first a checklist including age, gender, biochemical factors, ACI and the duration of dialysis and history of cardiovascular diseases in patients were collected. Statistical analysis was performed using SPSS Version. 20. 

Quantitative variables were shown as mean ± SD. The Kolmogorov-Smirnov test was used to evaluate the normality of distribution of quantitative data. Chi-square test was used to compare the ACI with respect to gender, duration of dialysis, and history of cardiovascular disease. To investigate the relationship between ACI and other chemical factors, Kruskal Wallis test for nonparametric variables and One Way ANOVA for analysis of variables with normal distribution were used. A p-value less than 0.05 was considered significant.

## Results

**Figure 2:**
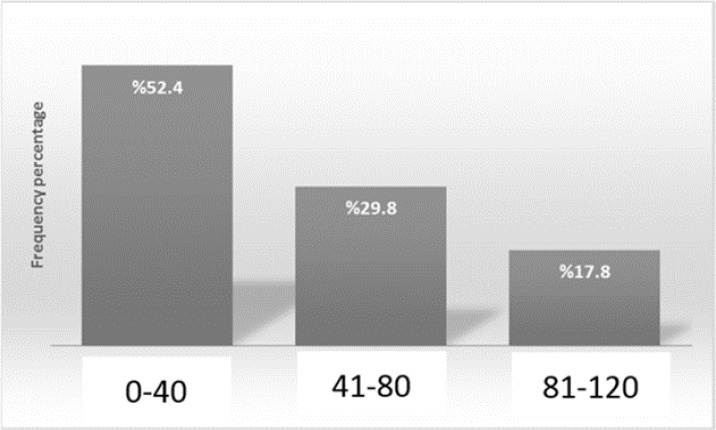
Frequency percentage of ACI different values

A total of 84 patients were studied. The average age of participants was 50.15±17.03 years (range of 18-83 years). 46 (54.8%) were men and 38 (45.2%) were women. 29 (34.5%) patients were on chronic dialysis less than 3 years and 55 (65.5%) patients for more than 3 years. 44 (52.4%) had no history of cardiovascular events and 40 (47.6%) had a history of cardiovascular or cerebrovascular events. The average of paraclinical parameters were: calcium (8.67+0.97 mg/dl), phosphorous (4.04+1.37 mg/dl) with normal distribution but alkaline phosphates (median 350 U/L), intact PTH (median 322 Pg/ml) and C-reactive protein (median 5.50 mg/L) with abnormal distribution.  Most of patients with a frequency of 44 (52.4%) cases had an ACI score between 0-40 (fig 2).

As seen in table 1, two variables (duration of dialysis and history of cardiovascular events, and strokes) had a significant relationship with ACI. But this relationship was not seen with gender. 

Table 2 shows the relationship between the ACI and the duration of dialysis and a history of vascular events based on gender.

**Table 1:** Relationship between ACI and gender, duration of dialysis, and history of vascular events

**Variable**	**ACI score**			**P-value** *
	**0-40** **N(%)**	**41-80** **N(%)**	**81-120** **N(%)**	
**Gender**				
Male Female	25(54.3) 19(50.0)	14(30.4) 11(28.9)	7(15.2) 8(21.1)	0.78
**Duration of dialysis**				
< 3 years > 3 years	23(79.3) 21(38.2)	3 (10.3) 22(40.0)	3(10.3) 12(21.8)	0.001
**History of vascular events**				
Yes No	5 (12.5) 39(88.6)	20(50.0) 5 (11.4)	15(37.5) -	<0.0001

* P-value, chi-square test

Except for duration of dialysis in women, a significant association was seen with other parameters. Examining the relationship between ACI and serum biochemical factors, statistically significant correlation was observed between ACI and ALK-P (p=0.01) (table 3). 

Regarding the relationship between the ACI amount and biochemical in men and women, it was found that P factor in women has a significant relationship with ACI (p=0.01). ALK-P in men also had a significant relationship with ACI (p=0.02).

 The results of the relationship between ACI and biochemical factors are shown in details in table 4. No significant relationship was observed between ACI and serum biochemical factors based on dialysis duration (table 5).

**Table 2:** Relationship between ACI score in the duration of dialysis and history of coronary events based on gender

**Gender**			**ACI score**			**P-value** *
			**0-40** **N(%)**	**41-80** **N(%)**	**81-120** **N(%)**	
Male	Duration of dialysis	< 3 years > 3 years	17 (%77.3) 8 (%33.3)	2 (%9.1) 12 (%50.0)	3 (%13.6) 4 (%16.7)	0.004
Female		< 3 years > 3 years	6 (%85.7) 13 (%41.9)	1 (%14.3) 10 (%32.3)	- 8 (%25.8)	0.11
Male	history of vascular events	Yes No	4 (%16.7) 21 (%95.5)	13 (%54.2) 1 (%4.5)	7 (%29.2) -	< 0.0001
Female		Yes No	1 (%6.3) 18 (%81.8)	7 (%43.8) 4 (%18.2)	8 (%50.0) -	< 0.0001

* P-value, Chi-square test

**Table 3:** Relationship between ACI score and serum biochemical parameters

**Biochemical factors**	**ACI score**			**P-value** *
	**0-40**	**41-80**	**81-120**	
Calcium (mg/dl) (mean±SD)	8.57±0.83	8.82±1.16	8.81±1.05	0.50
Phosphorus(mg/dl) (mean±SD)	3.86±1.51	4.42±1.19	3.94±1.13	0.06
Alkaline phosphatase(U/L) median(MIN-MAX	300 (38-2630)	331 (166-1534)	520 (210-1450)	0.01
iPTH( pg/ml) median(MIN-MAX)	280 (16-1269)	339 (67-870)	360 (69-1389)	0.16
CRP(mg/L) median(MIN-MAX)	3 (2-86)	12 (2-68)	11.5 (2-83)	0.33

* P-value, One – way ANOVA test

**Table 4:** Relationship between biochemical parameters and ACI based on gender

**Variable**	**Gender**	**ACI score**			**P-value** *
		**0-40**	**41-80**	**81-120**	
Ca(mg/dl) (mean±SD)	Male	8.44±0.90	8.78±1.46	8.55±1.15	0.66
	Female	8.74±0.70	8.88±0.68	9.03±0.97	0.64
P(mg/dl) (mean±SD)	Male	4.16±1.69	4.32±1.38	4.10±1.30	0.84
	Female	3.48±1.18	4.54±0.93	3.80±1.04	0.01
iPTH( pg/ml) median(min-max)	Male	284(16-1269)	381.5(67-772)	360(190-507)	0.54
	Female	261(28-665)	327(125-870)	455.5(69-1389)	0.27
Alk.P(U/L) median(min-max)	Male	298(68-2630)	287(166-892)	540(391-698)	0.02
	Female	314(38-753)	450(226-1534)	478(210-1450)	0.17
CRP(mg/L) median(min-max)	Male	6 (2-78)	15.5 (2-55)	15 (2-71)	0.21
	Female	2 (2-86)	2 (2-68)	7 (2-83)	0.75

* P-value, Kruskal Wallis test

**Table 5:** Relationship between biochemical factors and ACI score based on dialysis duration

**Variables**	**Dialysis duration**	**ACI score**			**P value**
		**0-40**	**41-80**	**81-120**	
Ca(mg/dl) (mean±SD)	< 3 years	8.48±0.93	8.20±1.05	8.10±1.05	0.73*
	> 3years	8.66±0.71	8.91±1.17	8.99±1.02	0.58*
P(mg/dl) (mean±SD)	< 3years	3.63±1.31	3.70±0.17	3.56±1.00	0.79*
	> 3years	4.11±1.71	4.52±1.23	4.03±1.19	0.26*
iPTH( pg/ml) median(min-max)	< 3years	199 (28-1269)	320 (305-403)	317 (280-376)	0.65**
	> 3years	284 (16-1038)	353 (67-870)	381.5 (69-1389)	0.23**
Alk.P(U/L) median(min-max)	< 3years	294 (68-2630)	318 (266-549)	540 (488-541)	0.08**
	> 3years	343 (38-753)	366 (166-1534)	500 (210-1450)	0.23**
CRP(mg/L) median(min-max)	< 3years	6 (2-86)	2 (2-42)	15 (2-28)	0.74**
	> 3years	2 (2-86)	13 (2-68)	11.5 (2-83)	0.52**

* P-value : One way ANOVA test

** P-value : Kruskal Wallis test

## Discussion

In this study, the relationship between abdominal ACI and biochemical parameters, duration of dialysis, cardiovascular events or stroke and gender in chronic hemodialysis patients were studied. ACI in 40% of patients who were under dialysis for more than 3 years, was in the range of 41-80, while around 10% of patients with dialysis for less than 3 years, had a 41-80 ACI. Futhermore, the percentage of the ACI in 81-120 was higher in hemodialysis patients with over 3 years than dialysis patients with less than 3 years, and the relationship was statistically significant. On the other hand, the patients with a history of cardiovascular events had bigger ACI than patients with no history of the disease. So, 37.5% of patients with a history of cardiovascular events had the ACI between 81-120, but none of the patients with no history of cardiovascular events had the ACI between 81-120. 

With regard to gender, ACI in men was associated with duration of dialysis, but this relationship was not seen in women. 50% of men, who were under dialysis for more than 3 years, had the ACI between 41-80. In the case of history of coronary events, a highly significant correlation was observed with the ACI in men and women. In this study, ACI and mean alkaline phosphatase show a statistically significant relationship. In females, the group with ACI in the range of 41-80 has the highest phosphorus While the ACI in men in the range of 81-120 has the highest alkaline phosphatase.

First, we examined the level of the ACI in patients with dialysis duration of less than 3 years. It was observed that in patients with a longer history of dialysis, the ACI level was significantly higher. This may be a result of long hyperparathyroidism and the consequence of the destruction of the vessels. In studies by Litwin (2005), Goodman (2000), and Oh (2002), it was also found that vascular damage was more in patients with longer duration of dialysis (7-9). In a study by Block et al., (2005), it was concluded that vascular calcification occured in 65% of adult patients with chronic kidney disease who started dialysis. This trend increases with increasing duration of dialysis (10). Finally, the findings of this study are consistent with the results of the above studies.

Next step, we studied the level of the ACI in patients with and without a history of cardiovascular events. The results showed that a higher level of ACI was observed in patients with vascular events and this difference was statistically significant. In studies done before, the relationship between ACI and cardiovascular events were never directly and independently investigated. But previously, it was seen that on the one hand in chronic hemodialysis patients with positive history of vascular events, the carotid intima-media thickness (CIMT) is also higher (11, 12) while a positive strong relationship was seen between CIMT and ACI (2, 13). By summing up these results, a relationship between ACI and vascular events was shown definitely in our study, it appears for the first time that such a relationship was directly and independently sought and as expected, a strong positive correlation was also found. The study examined the association between ACI and biochemical factors such as calcium, phosphorus, parathoromone, alkaline phosphatase and C - reactive protein. We found that ACI is associated with alkaline phosphatase. Then, the relationship between ACI and biochemical factors such as calcium, phosphorus, PTH, alkaline phosphatase and C - reactive protein based on gender was examined.

Several studies showed that the high alkaline phosphatase is a strong risk factor for cardiovascular and non-cardiovascular mortality in dialysis patients (14). In a study by Regidor et al., it was found that patients with alkaline phosphatase more than 120 U/L was associated with a mortality rate increased of 1.25 times (15). Beddhu et al., also found that higher alkaline phosphatase, independent of bone metabolism parameters and liver enzymes, increases mortality in hemodialysis patients. (16) Compared to ACI and ALK-P according to gender, a significant relationship was observed between ACI and ALK-P in men, but we did not find a reason to this relationship. When the association between ACI and P was analyzed based on gender, a relationship was found between these two parameters in female patients that did not exist in men . No logical explanation was found for this difference .In the above 2 cases, it could not be explained and justified, including other parameters such as underlying diseases, socioeconomic status and habits of patients though their ability for regular and timely follow-up of treatment may help.

In conclusions patients with longer duration of dialysis, and patients with a history of cardiovascular events and stroke, ACI level was significantly higher. There is a strong relationship between Alkaline phosphatase level and ACI in men and between phosphorus and ACI in women.

**The limitations and strengths of this study: **As far as our search is concerned, there are few studies in which the relationship between ACI index on the one hand and biochemical factors, the duration of dialysis and history of vascular events on the other hand have been studied. It seems that ACI can be used as a useful indicator to predict the occurrence of vascular events in ESRD patients. The method used in this study for ACI categorization (0-40, 41-80, 81-120) in ESRD patients has been used just one time in Japan (2) and repeated here now. Healthy people in the ACI categorization method which was used in this study had not been investigated. Consequently, actually there was no control group against case group (ESRD patients).
